# Respiratory syncytial virus: an under-recognized healthcare-associated infection

**DOI:** 10.1017/ice.2025.88

**Published:** 2025-06

**Authors:** Erin B. Gettler, H. Keipp Talbot, Yuwei Zhu, Danielle Ndi, Edward Mitchel, Tiffanie M. Markus, William Schaffner, Bryan Harris, Thomas R. Talbot

**Affiliations:** 1 Division of Infectious Diseases, Department of Medicine, Duke University Medical Center, Durham, NC, USA; 2 Department of Health Policy, Vanderbilt University Medical Center, Nashville, TN, USA; 3 Division of Infectious Diseases, Department of Medicine, Vanderbilt University Medical Center, Nashville, TN, USA; 4 Department of Biostatistics, Vanderbilt University Medical Center, Nashville, TN, USA

## Abstract

**Objective:**

Prior reports of healthcare-associated respiratory syncytial virus (RSV) have been limited to cases diagnosed after the third day of hospitalization. The omission of other healthcare settings where RSV transmission may occur underestimates the true incidence of healthcare-associated RSV.

**Design:**

Retrospective cross-sectional study.

**Setting:**

United States RSV Hospitalization Surveillance Network (RSV-NET) during 2016–2017 through 2018–2019 seasons.

**Patients:**

Laboratory-confirmed RSV-related hospitalizations in an eight-county catchment area in Tennessee.

**Methods:**

Surveillance data from RSV-NET were used to evaluate the population-level burden of healthcare-associated RSV. The incidence of healthcare-associated RSV was determined using the traditional definition (i.e., positive RSV test after hospital day 3) in addition to often under-recognized cases associated with recent post-acute care facility admission or a recent acute care hospitalization for a non-RSV illness in the preceding 7 days.

**Results:**

Among the 900 laboratory-confirmed RSV-related hospitalizations, 41 (4.6%) had traditionally defined healthcare-associated RSV. Including patients with a positive RSV test obtained in the first 3 days of hospitalization and who were either transferred to the hospital directly from a post-acute care facility or who were recently discharged from an acute care facility for a non-RSV illness in the preceding 7 days identified an additional 95 cases (10.6% of all RSV-related hospitalizations).

**Conclusions:**

RSV is an often under-recognized healthcare-associated infection. Capturing other healthcare exposures that may serve as the initial site of viral transmission may provide more comprehensive estimates of the burden of healthcare-associated RSV and inform improved infection prevention strategies and vaccination efforts.

## Introduction

Respiratory syncytial virus (RSV) is a common cause of acute lower respiratory tract infections among children^1^ and is increasingly recognized as a cause of clinically significant infections in adults, especially among those with cardiopulmonary conditions, advanced age, or immunocompromise.^2,3^ RSV results in nearly 160,000 hospitalizations and approximately 9,500–12,700 deaths among adults 65 years and older annually in the United States.^4^ Despite the associated morbidity and mortality, RSV is often under-recognized as an epidemiologically important pathogen in healthcare-associated infections; however, relative to influenza, RSV is more often hospital-acquired^5,6^ and associated with a higher risk of mechanical ventilation, prolonged length of hospital admission, and death.^2,3,5,7,8^

Most published reports of healthcare-associated RSV describe the experience of outbreaks at single institutions.^9–12^ Of the few published estimates of healthcare-associated RSV, most are limited to cases acquired in the hospital^13^ or restricted to pediatric patients.^14,15^ As described in regards to healthcare-associated influenza,^16^ omission of pre-admission healthcare exposures (e.g., residence in a post-acute care facility, recent admission to an acute care facility, or key ambulatory healthcare visits) that may serve as the initial location of viral transmission may underestimate the burden of healthcare-associated RSV. Using surveillance definitions previously described,^16^ this study aimed to provide a population-level, comprehensive assessment of healthcare-associated RSV to better understand the burden of RSV and the frequency of non-acute care healthcare exposures that result in hospitalization for RSV.

## Methods

The RSV Hospitalization Surveillance Network (RSV-NET) is 1 of 3 platforms supported by the Centers for Disease Control and Prevention that conducts population-based surveillance for hospitalizations associated with laboratory-confirmed RSV, COVID (COVID-NET), and influenza (FluSurv-NET) in acute care hospitals within the 12 states participating in the Emerging Infections Program.^17,18^ Within RSV-NET, persons of any age residing within the defined catchment area admitted between October and April of every year with a positive RSV test collected within 14 days prior to or during the hospitalization are included. Medical history, clinical course, medical interventions, and outcomes are obtained through chart abstraction performed by trained personnel. In this study, persons meeting the RSV-NET case definition admitted between 2016–2019 and residing in an eight-county catchment area in middle Tennessee (i.e., Cheatham, Davidson, Dickson, Robertson, Rutherford, Sumner, Williamson, and Wilson counties) were included. Laboratory confirmation was defined as a positive RSV test by molecular assay or rapid antigen testing.

**Figure 1.**
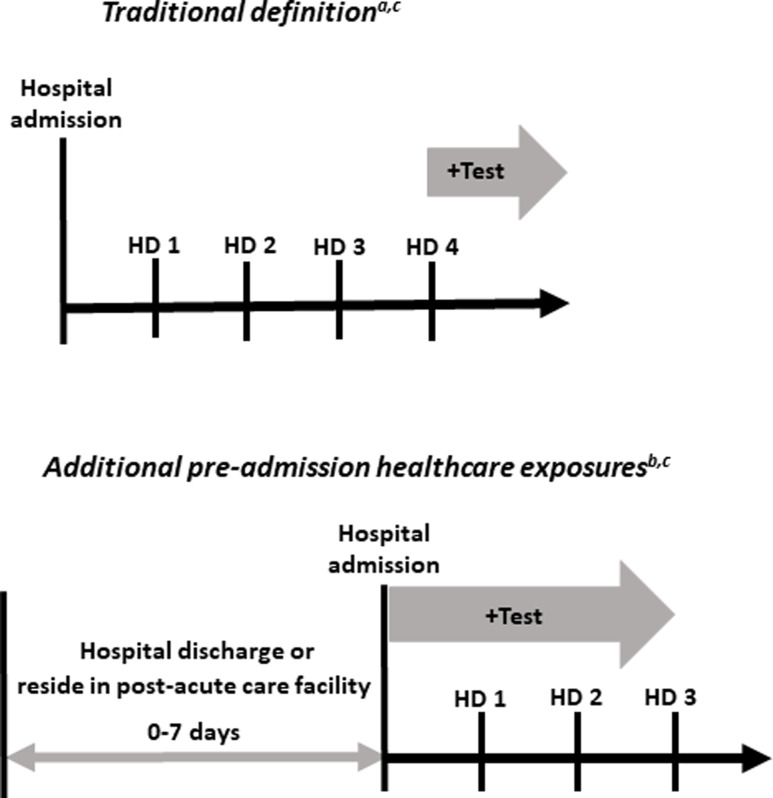
Traditional definition of healthcare-associated RSV and additional pre-admission healthcare exposures. *Note*: HD, hospital day. ^a^”RSV”-related hospitalizations with a positive test after day 3 were included in the traditional definition. ^b^”RSV”-related hospitalizations with a positive test in the first 3 days of admission among patients directly transferred from a post-acute care facility or discharged from a non-RSV-related acute care hospitalization in the 7 days preceding the index admission were included in the additional pre-admission healthcare exposure category. ^c^Cases meeting the traditional definition and those captured by the addition of the pre-admission healthcare exposures were included in the expanded definition of healthcare-associated RSV.

Using definitions previously applied in the evaluation of healthcare-associated influenza,^16^ the “traditional” definition of healthcare-associated RSV was defined as cases that occurred in persons with a positive RSV test after day 3 of the index hospitalization. In order to include other pre-admission healthcare settings in which RSV virus exposure and transmission may have occurred, persons with a positive RSV test collected in the first 3 days of hospitalization who were either (1) transferred from a post-acute care facility or (2) discharged from an acute care facility for a non-RSV illness in the 7 days preceding the index RSV admission were identified (Figure 1). These “additional healthcare exposures” combined with the cases meeting the “traditional” definition collectively represent the more comprehensive “expanded” definition of healthcare-associated RSV. Cases transferred directly from skilled nursing facilities, inpatient rehabilitation facilities, long-term acute care hospitals, inpatient hospice, and mental health facilities were included. Recent acute care hospital discharges were ascertained from RSV-NET and the Tennessee Department of Health Hospital Discharge Data System, as previously described.^16^

Cases defined as healthcare-associated RSV by the traditional and expanded definitions were expressed as a percentage of the total laboratory-confirmed RSV-related hospitalizations. Additionally, the proportion of RSV cases attributed to each type of healthcare exposure (i.e., index hospitalization, association with post-acute care transfer, or association with a recent acute care admission) were calculated. Crude incidence rates per 100,000 population stratified by type of healthcare exposure were calculated using the number of cases meeting the definitional criteria as the numerator and the population within the predefined catchment area, as recorded by the National Center for Health Statistics’ vintage bridged-race postcensal population estimates,^19^ between 2016 and 2019 as the denominator. Categorial variables were compared using *χ* ^2^ tests. Analyses were conducted in Stata (Release 18, StataCorp LLC, College Station, TX). This study was approved by the Vanderbilt University Medical Center and Tennessee Department of Health Institutional Review Boards.

## Results

A total of 900 laboratory-confirmed RSV-related hospitalizations were captured across 3 respiratory seasons in the surveillance catchment area. Age distribution was bimodal with the majority of cases concentrated in children less than 18 years of age (31%) and adults 65 years of age or older (40%, Table 1). Most adults had one or more underlying medical condition (64%). In the total cohort, over 20% of patients admitted with RSV required ICU-level care and had a median hospital length of stay of 4 days (IQR, 2–6 days). Of these RSV-related hospitalizations, 41 (4.6%) had traditionally defined healthcare-associated RSV. Relative to the total cohort, these patients had a longer median length of stay (9 days, IQR 6–22) and a higher proportion required ICU admission (32% vs 21%, *P*-value 0.11), although this was not statistically significant.

**Table 1.** Characteristics of patients with RSV-related hospitalization, RSV-net, 2016–2019

Characteristic	All RSV Hospitalizations (Community and Healthcare-Associated) N = 900 (%)	Healthcare-Associated RSV as Defined Using Traditional Definition N = 41 (%)	Healthcare-Associated RSV Among Patients with a Pre-Admission Healthcare Exposure N = 95 (%)	*P* value^a^
**Sex**				0.237
Male	384 (43%)	20 (49%)	36 (38%)	
Female	516 (57%)	21 (51%)	59 (62%)	
**Age group, years**				0.702
0–17	284 (31%)	7 (17%)	18 (19%)	
18–49	80 (9%)	2 (5%)	9 (9%)	
50–64	180 (20%)	13 (32%)	23 (24%)	
≥65	356 (40%)	19 (46%)	45 (47%)	
**Age, median years (IQR)**	59 (2–73)	60 (55–79)	63 (43–74)	
**Race** ^b^				0.308
American Indian/Alaska Native	1 (<1%)	0	0	
Asian/Pacific Islander	17 (2%)	1 (2%)	0	
Black	160 (18%)	4 (10%)	19 (20%)	
Multiple races	4 (<1%)	0	1 (1%)	
White	654 (72%)	33 (80%)	69 (73%)	
Unspecified, Not reported	64 (7%)	3 (7%)	6 (6%)	
**Ethnicity** ^b^				0.802
Hispanic or Latino	35 (4%)	1 (2%)	4 (4%)	
Non-Hispanic or Latino	736 (82%)	35 (85%)	77 (81%)	
Unspecified, Not reported	129 (14%)	5 (12%)	14 (15%)	
**Transferred from another hospital**	32 (4%)	1 (2%)	11 (12%)	0.085
**Children <18 years with 1 or more underlying medical conditions**	38 (13%)	0	3 (17%)	0.25
**Adults ≥18 years with 1 or more underlying medical condition**	392 (64%)	24 (71%)	60 (78%)	0.407

a Healthcare-associated RSV among patients with pre-admission healthcare exposures was compared to healthcare-associated RSV as defined by the traditional definition using χ^2^ tests.

b Self-reported race and ethnicity as captured in the electronic medical record. Ethnicity is reported separately as race and ethnicity are not mutually exclusive.

**Figure 2.**
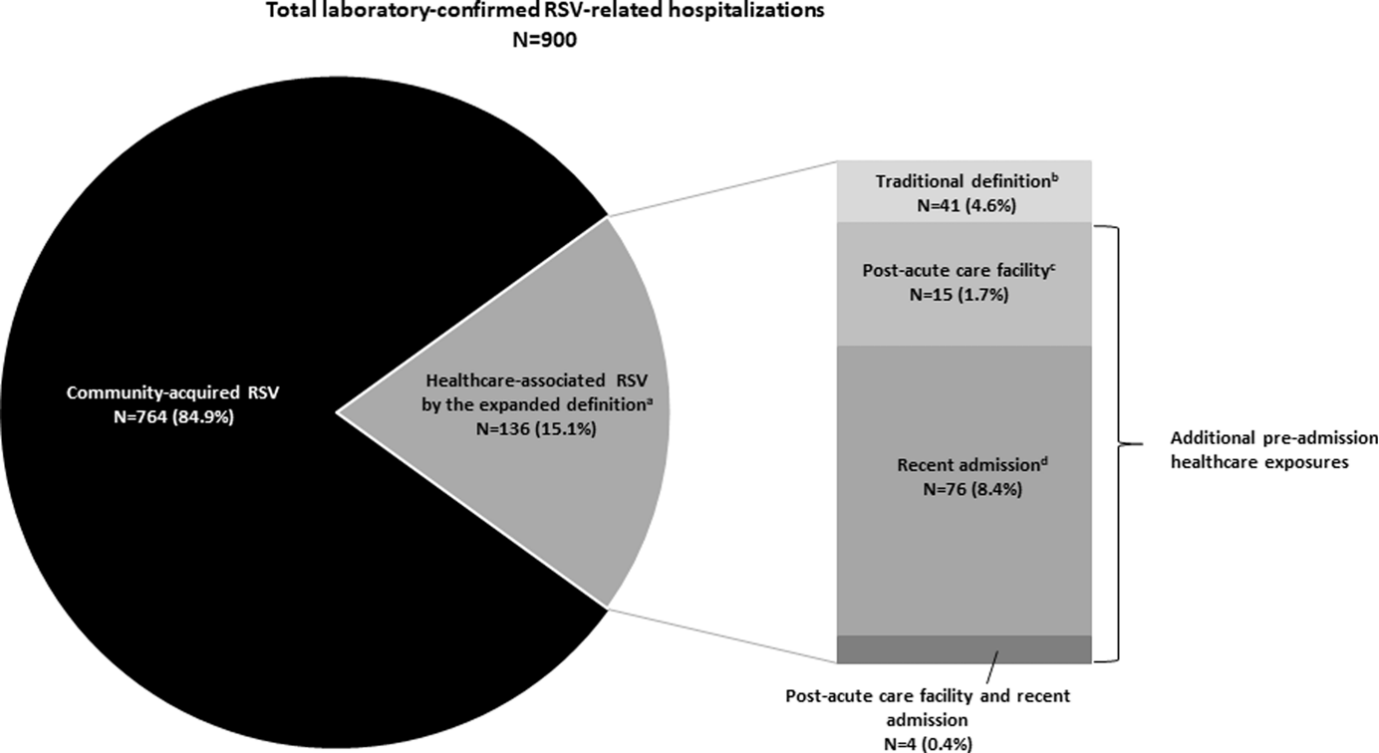
Proportion of RSV-related hospitalizations by type of healthcare exposure, RSV-net, 2016–2019. ^a^Cases meeting the traditional definition and those captured by the additional pre-admission healthcare exposures were included in the expanded definition of healthcare-associated RSV. ^b^RSV-related hospitalizations with a positive test after hospital day 3 were included in the traditional definition. ^c^RSV-related hospitalizations with a positive test in the first 3 days of admission in patients directly transferred from a post-acute care facility. ^d^RSV-related hospitalizations with a positive test in the first 3 days of admission in patients discharged from a non-RSV-related acute care hospitalization in the 7 days preceding index admission.

The inclusion of persons with a positive RSV test obtained in the first 3 days of hospitalization with a pre-admission healthcare exposure identified an additional 95 cases (10.6% of all RSV-related hospitalizations). Of these additional cases, 15 (1.7%) were transferred from a post-acute care facility, 76 (8.4%) had a recent acute care hospital admission in the preceding 7 days, and 4 (0.4%) had both a recent acute care hospital admission and contact with a post-acute care facility (Figure 2). Similar to the cases defined using the traditional definition, the cohort identified with these additional healthcare exposures had a longer median length of hospital stay (6 days, IQR 3–12) and greater proportion with an ICU admission compared to the total cohort (35% vs 21%, *P*-value 0.003). After including the additional healthcare exposures into the expanded definition, the proportion of laboratory-confirmed RSV-related hospitalizations that were possibly healthcare-associated increased from 4.6% to 15.1%.

Among all RSV-related hospitalizations, the crude rate of traditionally defined healthcare-associated RSV was 0.8 per 100,000 (with an annual seasonal range of 0.6–1.1) (Supplemental Table 1). This rate increased to 2.65 per 100,000 (annual seasonal range 1.9–3.6) when the more comprehensive expanded definition of healthcare-associated RSV was used. Relative to the traditional definition, the incidence rate ratio of healthcare-associated RSV by the expanded definition was over 3-fold higher (3.3; [95% CI, 2.3–4.7]).

## Discussion

RSV is increasingly recognized as an epidemiologically important pathogen among children and adults, especially after the surge in RSV cases following the COVID-19 pandemic. Yet, RSV is often omitted from estimates of healthcare-associated viral respiratory infections. Few studies provide population-level surveillance of healthcare-associated RSV, and these reports are often limited to pediatric populations^14,15^ or traditionally defined cases with hospital-onset of symptoms or positive diagnostic testing after 3 to 4 days of hospitalization.^13^ This study addresses several of these gaps. Using a population-level surveillance system of laboratory-confirmed RSV among hospitalized patients over 3 years in middle Tennessee, this study illustrates the incidence of healthcare-associated RSV among all ages, including older adults, and the potential underestimation of healthcare-associated RSV by omitting pre-admission healthcare exposures.

As demonstrated in a systematic review by French et al., the risk of nosocomial RSV transmission can be substantial^20^ and occur in multiple healthcare settings.^9–12,21^ Improved understanding of how different healthcare settings may contribute to healthcare-associated RSV has important implications for infection control practices. Outpatient and long-term care facilities often present unique challenges to the general application of infection prevention practices that differ from those used in inpatient facilities.^22,23^

While inclusion of other healthcare settings is important for comprehensive surveillance, transmission of RSV within acute care hospitals remains a significant concern. In a prospective study of patients with either community- or healthcare-associated influenza-like illness, Kestler et al. demonstrated that a significant number of adults actually have RSV, and RSV was more frequently healthcare-acquired than influenza.^5^ The authors have previously published a comparison of the rates of healthcare-associated influenza using the novel surveillance definitions presented in this study.^16^ Using these data, and similar to Kestler et al., we found a greater proportion of healthcare-associated RSV met the traditional definition compared to influenza. Additionally, those with healthcare-associated RSV required more ICU-level care and had a longer length of stay, findings that have been illustrated in prior studies.^2,3,7,8^ These findings emphasize the importance of improved clinician awareness and early detection of RSV in order to implement respiratory isolation and horizontal infection control measures to lessen further nosocomial transmission.

This study has several possible limitations. First, categorizing cases as community- or healthcare-associated based on the date of test positivity may lead to misclassification, particularly without chart review to correlate timing of symptom onset relative to test positivity. The delay between admission and date of test positivity used in the traditional definition is consistent with the median incubation period for RSV.^24,25^ While still near the 95^th^ percentile of the incubation period for RSV,^25^ the inclusion of cases that occurred within 7 days of discharge from an acute care facility may misrepresent some cases that were actually community-acquired. Conversely, not all potential pre-admission healthcare exposures were captured in the expanded definition, possibly leading to misattribution of cases as community-associated if the actual viral transmission occurred in these healthcare settings. Second, only RSV cases that required hospitalization were captured. Additionally, while this study does present a population-level estimate of the incidence of RSV, the catchment area is limited to a region in middle Tennessee and specific findings may not be widely generalizable. However, the novel strategy introduced of applying a more comprehensive definition of healthcare-associated RSV could be readily applied to other geographical regions for similar study. Lastly, while these study results support the need for comprehensive and systematic surveillance of healthcare-associated RSV, there are barriers to capturing these other exposures accurately as part of a routine surveillance program. Mandatory reporting of hospitalized patients with laboratory-confirmed RSV, as well as influenza and COVID-19, to the Centers for Medicare and Medicaid Services began in November 2024 and may help fill some of these gaps.

In summary, RSV remains an under-recognized nosocomial infection. Restricting surveillance of healthcare-associated RSV to only those cases possibly acquired during the current acute care admission likely underestimates the true burden of cases. Inclusion of RSV in respiratory virus surveillance methods could improve the understanding of RSV transmission and inform infection prevention strategies and clinical practice, including more widespread use of RSV immunization.

## Supporting information

Gettler et al. supplementary material Gettler et al. supplementary material

## Supplementary material

To view supplementary material for this article, please visit https://doi.org/10.1017/ice.2025.88
