# Supplementary material for: Respiratory syncytial virus: an under-recognized healthcare-associated infection
Source: Infect Control Hosp Epidemiol. 2025 May 16;46(6):611–5. doi: 10.1017/ice.2025.88 (PMC12169951; doi:10.1017/ice.2025.88)
Supplement: Gettler et al. supplementary material [file S0899823X25000881sup001.docx]

**Supplemental Table 1. Population-level Incidence and Incidence Rate Ratio (IRR) of Healthcare-Associated RSV by Traditional and Expanded Definitions**

|  | **Incidence (per 100,000 population) by respiratory virus season and total study period** | | | | IRR^a^ (IQR) |
| --- | --- | --- | --- | --- | --- |
|  | 2016-2017 | 2017-2018 | 2018-2019 | Total Study Period |  |
| Healthcare-Associated RSV by Traditional Definition | 0.7 | 0.6 | 1.1 | 0.8 | ___ |
| Healthcare-Associated RSV by Expanded Definition | 1.9 | 2.4 | 3.6 | 2.7 | 3.3 (2.3-4.7) |

^a^Healthcare-associated RSV using the traditional definition was used as the reference for IRR.
